# Tumour budding at the deepest invasive margin correlates with lymph node metastasis in submucosal colorectal cancer detected by anticytokeratin antibody CAM5.2

**DOI:** 10.1038/sj.bjc.6602927

**Published:** 2006-01-10

**Authors:** S Kazama, T Watanabe, Y Ajioka, T Kanazawa, H Nagawa

**Affiliations:** 1 Department of Surgery, Division of Surgical Oncology, Faculty of Medicine, The University of Tokyo, 7-3-1 Hongo, Bunkyo-Ku, Tokyo 113-8655, Japan; 2 First Department of Pathology, School of Medicine, Niigata University, 1-757 Asahimachi-dori, Niigata city 951-8510, Japan

**Keywords:** tumour budding, micrometastasis, isolated tumour cells, CAM5.2, D2-40

## Abstract

In the past few years, tumour budding at the invasive margin has been reported as a new risk factor for lymph node metastasis in advanced colorectal cancers, but it is sometimes difficult to detect tumour budding in submucosal colorectal cancer by haematoxylin and eosin staining. We immunohistochemically examined tumour budding at the deepest invasive margin of 56 surgically resected submucosal colorectal carcinomas using anticytokeratin antibody CAM5.2, furthermore checked by AE1/AE3, and determined the relation between tumour budding and clinicopathological factors. Moreover, we used the monoclonal antibody D2-40 for immunohistochemistry to detect lymphatic involvement. Tumour budding was detected in 42 cases (75.0%), and the budding-positive group showed a significantly higher rate of lymph node metastasis (including isolated tumour cells) (16/42 *vs* 0/14; *P*=0.004) than the budding-negative group. The sensitivity and negative predictive value of tumour budding alone for lymph node metastasis were superior to those of lymphatic invasion alone. Furthermore, the specificity and positive predictive value of the combination of either lymphatic invasion or tumour budding were superior to those of lymphatic invasion alone. Tumour budding detected immunohistochemically by using CAM5.2 is a newly found risk factor for lymph node metastasis and may help to avoid oversurgery in the future.

Recently, endoscopic polypectomy or endoscopical mucosal resection has been performed extensively for the treatment of colorectal adenomas, and intramucosal and submucosal carcinomas (Williams *et al*, 2000; Kudo *et al*, 2001; Tung *et al*, 2001). However, it has been reported that lymph node metastasis is present in about 10% of submucosal carcinomas (Wilcox *et al*, 1986; Muto *et al*, 1991). Therefore, if the polypectomised specimen is revealed to be a submucosal carcinoma, additional surgical resection of the colon or rectum with lymph node dissection is recommended. However, if additional surgery is performed for all submucosal carcinomas after endoscopical resection, only 10% of patients benefit from dissecting surgery for lymph node metastasis. In other words, about 90% of the patients undergo unnecessary surgery, since these patients do not have any lymph node metastasis. If the presence of lymph node metastasis can be accurately predicted from the polypectomised specimen, oversurgery can be avoided. In order to select those patients who are at a high risk for lymph node metastasis, lymphatic involvement has been considered a risk factor in polypectomised submucosal carcinoma (Morson *et al*, 1977; Haggitt *et al*, 1985; Muto *et al*, 1991). However, it is difficult to identify a lymphatic involvement by cancer cells with routine haematoxylin and eosin (H.E) staining and both the sensitivity and the positive predictive value of this risk factor for lymph node metastasis remain low. Studies of advanced colorectal cancers have recently demonstrated that tumour budding at the invasive margin is a risk factor for lymph node metastasis (Morodomi *et al*, 1989; Hase *et al*, 1993; Ono *et al*, 1996; Goldstein and Hart, 1999; Okuyama *et al*, 2002; Ueno *et al*, 2002; Tanaka *et al*, 2003; Park *et al*, 2005). However, so far there have been few studies that have examined tumour budding in submucosal carcinomas with respect to lymph node metastasis (Makino *et al*, 2000; Masaki *et al*, 2001b; Ueno *et al*, 2004). Pathologically tumour budding is defined as a single cell or a small cluster of cells away from the tumour mass. It is sometimes difficult, therefore, to detect tumour budding by examining the polypectomised specimen with H.E alone. Cytokeratin is a component of the cytoskeleton of the epithelial cells that is not present in the normal submucosal layer and normal lymph nodes.

Therefore, in the present study, we used not only H.E but also immunohistochemical staining CAM5.2, furthermore checked by AE1/AE3, to make a more accurate diagnosis of tumour budding and D2-40 to detect lymphatic involvement. It has recently been reported that CAM5.2 antibody is useful for detecting lymph node micrometastasis or isolated tumour cells (ITCs) in various gastrointestinal and biliary cancers (Oberg *et al*, 1998; Cai *et al*, 1999; Yokoyama *et al*, 1999; Yasuda *et al*, 2002; Tojima *et al*, 2003).

Therefore, we also examined the lymph node metastasis (including micrometastasis and ITCs) of submucosal cancer using CAM5.2. The monoclonal antibody D2-40, which has been developed against a testicular oncofetal antigen, has been demonstrated to recognise lymphatic vessels in the breast, colonic carcinoma and head and neck squamous cell carcinoma (Marks *et al*, 1999; Kahn and Marks, 2002; Fogt *et al*, 2004; Franchi *et al*, 2004).

The aim of the present study is to examine whether tumour budding could be a newly found risk factor for lymph node metastasis in submucosal carcinoma. Furthermore, we examined the relation between tumour budding and lymph node micrometastasis and ITCs by using CAM5.2. To the best of our knowledge, this is the first study that has examined tumour budding in submucosal colorectal carcinoma by CAM5.2 immunohistochemistry with respect to lymph node metastasis.

## MATERIALS AND METHODS

### Specimens

We examined 56 surgically resected submucosal colorectal adenocarcinomas from the surgical pathology files of the Department of Surgical Oncology, The University of Tokyo, Tokyo, Japan from March 1990 to June 2001. The resected specimens were immediately fixed in 10% buffered formalin and the entire tumours were cut into step-wise sections and embedded in paraffin. Pathological diagnoses of the primary lesions were made by H.E, and the presence of venous and lymphatic permeation was assessed in greater detail by the methods described below. In order to confirm the deepest points of invasion and the presence of venous and lymphatic permeation, one to 17 step-wise sections were cut from each primary tumour.

### Histological examination

**Figure 1:**
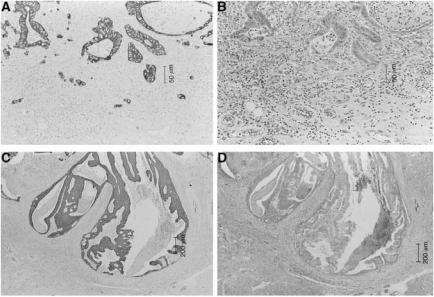
(**A**) Immunohistochemical staining of tumour budding using an anticytokeratin antibody, CAM5.2 (original magnification, × 50). (**B**) Tumour budding at the invasive margin, using H.E (original magnification, × 50). (**C**) Immunohistochemical staining of a case without tumour budding using an anticytokeratin antibody, CAM5.2 (original magnification, × 10). (**D**) A case without tumour budding at the invasive margin, using H.E (original magnification, × 10).

Two 3-*μ*m-thick sections for CAM5.2 (Becton Dickinson, San Jose, CA, USA) (diluted 1 : 3) and AE1/AE3 (Dako, Kyoto, Japan) (diluted 1 : 150) immunostaining were cut from the deepest invasive specimen in order to examine the budding at the deepest invasive margin (Figure 1).

**Figure 2:**
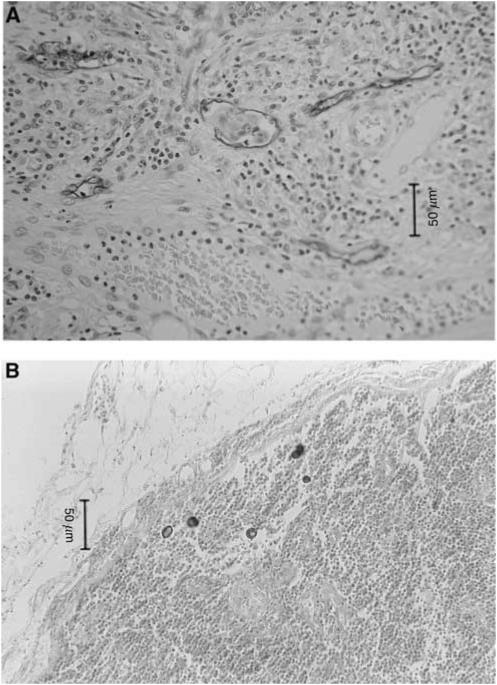
(**A**) Lymphatic involvement by immunostaining with anti-human lymphatic endothelial cell D2-40 (original magnification, × 50). (**B**) Isolated tumour cells from colorectal carcinoma in a lymph node. Immunohistochemical staining using an anticytokeratin antibody, CAM5.2 (original magnification, × 50).

For the examination of venous and lymphatic permeation, three 3-*μ*m-thick sections were prepared. The first of the three 3 *μ*m sections was used for H.E, the next for Victoria blue elastic fibre staining to visualise the elastic fibres of the venous wall and the last was used for immunostaining with anti-human lymphatic endothelial cell D2-40 (Signet, Dedham, MA, USA) (diluted 1 : 100) to visualise the endothelial cells of the lymphatic channels (Figure 2A).

### Lymph nodes

Lymph nodes were routinely examined by one section through the centre of each lymph node. Two experienced pathologists independently examined all sections and confirmed the absence of visible metastatic deposit in all lymph nodes. A total of 628 lymph nodes were dissected from the 56 patients. The average number of lymph nodes dissected per patient was 11.2 (range, 1–60 nodes). For micrometastasis and ITCs analysis, we examined three serial sections, each 10 *μ*m thick (with an aggregate thickness of 30 *μ*m), from each of the paraffin blocks of the lymph nodes, which were used for CAM5.2 immunohistochemical examination as described below (Figure 2B).

### D2-40, CAM5.2 and AE1/AE3 immunohistochemical staining

The streptavidin–biotin (SAB) immunoperoxidase method was used. For D2-40 staining, the sections were deparaffinised with xylene and dehydrated with 98% ethanol, placed in 0.01 M sodium citrate buffer (pH 6.0), and heated in a microwave oven for three 7-min cycles (500 W). After washing twice in phospahte-buffered saline (PBS), endogenous peroxidase activity was inhibited by incubation with 0.3% hydrogen peroxide in methanol for 20 min. After three washes in PBS, nonspecific reaction was blocked by incubation at room temperature. Reagents for the subsequent step, biotinylated rabbit anti-mouse immunogloblin and SAB complex, supplied commercially (Histfine SAB-PO(M) kit, Nichirei, Tokyo, Japan) were used. The sections were incubated with anti-D2-40 antibody overnight at 4°C. Colour was then developed with diaminobenzidine solution. The sections were then lightly counterstained with a cocktail of Mayer’s/Lillie–Mayer’s haematoxylin and mounted. For CAM5.2 and AE1/AE3 staining, the sections were deparaffinised with xylene and dehydrated with 98% ethanol, and treated with 0.1% trypsin (Sigma Chemical Company, St Louis, MO, USA) in 0.1% calcium chloride (pH 7.8) at 37°C for 20 min before immunostaining; they were treated in the same way as with D2-40 staining, and counterstained with methyl green and mounted.

### Definition of tumour budding

Tumour budding refers to microscopic microtubular cancer nests and undifferentiated cancer cells at the invasive margin of the carcinomatous lesion, as proposed by Morodomi *et al* (1989) and Hase *et al* (1993). In this study, we examined this tumour budding by specimens stained by CAM5.2 immunohistochemistry, and checked by AE1/AE3 immunohistochemistry, because single tumour cells are more easily detected by staining with CAM5.2 than with H.E. Cases with budding were classified as the budding-positive group, and cases without budding as the budding-negative group.

### Definition of lymph node metastasis

Tumour deposits within lymph nodes were classified according to the revised guidelines set by American Committee on Cancer (AJCC) (Hermanek *et al*, 1999). The lymph nodes were classified as pN0, pN1 and pN2 according to the current AJCC criteria for colorectal cancer. Isolated tumour cells were single tumour cells or cell clusters that measured no greater than 0.2 mm and were detected by H.E and/or immunohistochemistry (pN0i− and pN0i+). Isolated tumour cells were included in the category of lymph node metastasis. Micrometastasis was diagnosed when the tumour nodule in the lymph node was smaller than 2 mm in diameter (pN1mi and pN2mi). Although reticular cells and plasma cells in lymph nodes can show staining for CAM5.2, these non-neoplastic cells were easily discriminated from ITCs by differing staining patterns, as described by Sasaki *et al* (1998).

### Statistical analysis

All statistical calculations were carried out with StatView-J 5.0 statistical software (SAS Institute, Cary, NC, USA). *χ*^2^ test and Student’s *t*-test were used to analyse data. A *P*-value <0.05 was considered to indicate statistical significance.

## RESULTS

### Clinicopathological features in submucosal colorectal carcinomas

The characteristics of the 56 tumours are shown in Table 1. Among seven cases with lymphatic involvement, six were diagnosed by H.E and one by D2-40 staining, this latter case was not detected by H.E. Among 31 cases with venous involvement, 20 were diagnosed by H.E and 11 by Victoria blue elastic fibre staining; the latter cases were not detected by H.E. Isolated tumour cells were detected in eight cases (14.3%), with the aid of immunohistochemistry (pN0i(+)) in seven cases and the aid of H.E (pN0i(−)) in one case. The lymph node from three of eight patients with pN1 nodal status at initial examination had metastasis measuring from 0.2 mm in diameter up to a maximum of 2 mm and could be reclassified as pN1mi. None of the pN1 patients were upstaged to pN2 as a result of encountering ITCs.

### Tumour budding in submucosal colorectal cancers

Tumour budding was detected in 42 cases (75.0%) by CAM5.2 immunohistochemistry, and all cases detected by CAM5.2 were also positive for AE1/AE3 immunohistochemistry. No case with tumour budding was newly detected by AE1/AE3 immunohistochemistry. Table 2 shows the correlation between tumour budding in the primary tumours of submucosal colorectal cancers and their clinicopathological features. The budding-positive group showed a significantly higher rate of lymph node metastasis (16/42 *vs* 0/14; *P*=0.004) than the budding-negative group. Moreover, the former group showed a higher rate of lymphatic involvement (7/42 *vs* 0/14; *P*=0.12) than the latter group, but not significantly. All 14 cases without tumour budding showed no lymphatic involvement or lymph node metastasis. There was no relation between tumour budding and venous involvement.

### Sensitivity, specificity and predictive value of lymphatic invasion and tumour budding for lymph node metastasis

The sensitivity, specificity and predictive value of lymphatic invasion, tumour budding and the combination of these two factors for lymph node metastasis are shown in Table 3. Sensitivity, specificity, positive predictive value and negative predictive value of lymphatic invasion alone for lymph node metastasis were 37.5, 97.5, 85.7 and 79.6%, respectively, whereas those of tumour budding alone (the combination of either lymphatic invasion or tumour budding) were 100, 65, 38.1 and 100% (43.7, 100, 100 and 81.6%), respectively.

## DISCUSSION

We were able to show that tumour budding is a newly discovered risk factor for lymph node metastasis in submucosal colorectal carcinoma. There is, therefore, a possibility that oversurgery for polypectomised submucosal colorectal carcinoma can be avoided by examining tumour budding.

Tumour budding is defined as anaplastic thin cell cords or individual free cells, microtubular cancer nests or undifferentiated cancer cells (Morodomi *et al*, 1989). This histological appearance is also expressed by various terms such as ‘sprouting’, ‘focal dedifferentiation’ and ‘budding’ (Morodomi *et al*, 1989; Ono *et al*, 1996). Tumour budding was first reported by Imai. Although he used the term ‘sprouting’ instead of ‘budding’, he showed that tumour with sprouting, regardless of the presence or absence of peritumour stromal invasion, have higher malignant potential in various cancers. Morodomi *et al* (1989) showed that tumour budding strongly correlated with lymphatic invasion and lymph node metastasis in rectal cancers. Hase *et al* (1993) reported that tumour budding was an important predictor for recurrence and poor prognosis in patients with colorectal cancers. Furthermore, some oncologists and pathologists showed that tumour budding was significantly associated with lymph node metastasis, local recurrence, distant metastasis and poor prognosis in advanced colorectal cancers (Ono *et al*, 1996; Goldstein and Hart, 1999; Okuyama *et al*, 2002; Ueno *et al*, 2002).

In previous reports, tumour budding was detected by using H.E specimens. However, considering that tumour budding is very small in size, since they are small cluster of cells or sometimes even a single cell away from the tumour mass, it is sometimes difficult to detect tumour budding by conventional pathological examination of the polypectomised specimen using this method. Therefore, in the present study, in addition to H.E, we examined tumour budding by not only CAM5.2 but also AE1/AE3 immunohistochemistry, which makes it much easier to detect a small number of cells. CAM5.2 is an antibody against cytokeratins 8 and 18, and AE1/AE3 is an antibody against cytokeratins 1–8, 10, 14–16 and 19, which are components of the cytoskeleton of the epithelial cells (Moll *et al*, 1982). CAM5.2 has been used to detect minute deposits of tumour cells. Recent studies have demonstrated the high sensitivity, high accuracy and cost effectiveness of this immunohistochemical method using CAM5.2 to detect lymph node micrometastasis or ITCs in various tumours originating from the stomach (Cai *et al*, 1999), colorectum (Oberg *et al*, 1998; Yasuda *et al*, 2002), gall bladder (Yokoyama *et al*, 1999) or bile duct (Tojima *et al*, 2003). Moreover, studies of lymph node ITCs in colorectal cancer by using CAM5.2 have shown that ITCs correlated significantly with poor prognosis (Haboubi *et al*, 1992; Isaka *et al*, 1999; Yasuda *et al*, 2002). Therefore, in the present study, we also examined ITCs in the lymph nodes with respect to tumour budding, using CAM5.2. To the best of our knowledge, this is the first report to demonstrate the relation between tumour budding and lymph node metastasis including ITCs in submucosal colorectal carcinoma by using CAM5.2 immunohistochemistry. At the present time, the main risk factor for lymph node metastasis in submucosal colorectal carcinoma is thought to be the presence of lymphatic involvement (Morson *et al*, 1977; Haggitt *et al*, 1985; Muto *et al*, 1991). However, the identification of lymphatic involvement by cancer cells on routine H.E has been difficult. In this study, we used the monoclonal antibody D2-40 to visualise the endothelium of lymphatic channel. However, in using this risk factor, sensitivity and positive predictive values of lymph node metastasis are low. In the present study, the sensitivity and negative predictive value of tumour budding alone were superior to those of lymphatic invasion alone, but the specificity and positive predictive value of tumour budding alone were not superior to those of lymphatic invasion alone. However, when both of these factors were combined, sensitivity and positive predictive value were not only 100%, but superior to those of lymphatic invasion alone. Furthermore, we were able to show that all cases without budding at the deepest invasive margin have no lymphatic invasion or lymph node metastasis. By defining the risk factor of lymph node metastasis as positive when either lymphatic invasion or tumour budding is present, we were able to demonstrate for the first time that all sensitivity, specificity, positive and negative predictive values for lymph node metastasis were superior to those of lymphatic invasion alone. These results show that tumour budding combined with lymphatic invasion is of great use as a risk factor for lymph node metastasis in polypectomised submucosal carcinoma. The higher negative predictive value of budding than that of lymphatic involvement is useful in clinical practice. The indicator for colectomy with lymph node dissection for previously polypectomised cancer may be determined based on budding. If the polypectomised specimen has no budding, additional colectomy, therefore, may not be indicated. Moreover, the higher positive predictive value of budding and lymphatic involvement than that of lymphatic involvement is useful, too. The combination of budding and lymphatic involvement may provide information useful for postoperative follow-up and for determining the need for adjuvant chemotherapy, even if the case was submucosal cancer.

In the past few years, important molecular events concerning with tumour budding, such as the gain and loss of adhesion molecules, secretion of proteolytic enzymes, reduction of tumour proliferation and mutation of tumour suppressor genes, have been examined. Sordat *et al* (2000) reported that budding cells in colorectal carcinomas underexpress the laminin-5 *α*3-subunit, while retaining expression of the *β*3- and *γ*2-subunits. Masaki *et al* (2001a, 2003) suggested that expression of the laminin-5 *γ*2-chain with or without matrilysin (MMP-7), or upregulation of CD44 variant 6 through nuclear *β*-catenin activation contributed to the formation of budding cell at the invasive front. Guzinska *et al* (2004) reported the activity of cathepsin B, which connect with proteolytic effect on basement membrane and intestinal stroma and has promotion role in carcinogenesis, correlated with tumour budding. Jung *et al* (2001) showed that tumour budding was associated with reduced proliferation, but with nuclear cyclin D1 expression. Moreover, Makino *et al* (2000) showed that tumour budding was significantly more frequent in p53-positive than p53-negative tumours, and Jass *et al* (2003) reported that the frequency of both budding and APC mutation was higher than that in microsatellite instability (MSI) high, hereditary nonpolyposis colorectal cancer, MSI low and MSI stable. They also emphasised that these findings indicate that tumour budding is a dynamic process under genetic control and not merely the result of architectural disruption caused by a host immune reaction at the tumour margin (Jass *et al*, 2003). The correlation between tumour budding and various molecular events may be helpful in our future understanding of the malignant potential of tumour budding in colorectal cancer, although more biological research is needed.

In conclusion, our results indicate that tumour budding correlates with lymph node metastasis in submucosal colorectal cancers, and that this parameter is a useful indicator of the risk of lymph node metastasis in such cancers. Detection of tumour budding by CAM5.2 immunohistochemistry may help to avoid oversurgery in the future. A new study with a larger number of cases, especially in a prospective and multicenter setting, is necessary.

## Figures and Tables

**Table 1:** Clinicopathological features in submucosal colorectal cancers

*Gender*	
Male	41 (73.2%)
Female	15 (26.8%)

Mean age (years)±s.d.	62.8±10.5
Size (mm)±s.d.	22.8±10.5

*Location*	
Caecum	1 (1.8%)
Ascending colon	7 (12.5%)
Transverse colon	7 (12.5%)
Descending colon	3 (5.4%)
Sigmoid colon	24 (42.9%)
Rectum	14 (25.0%)

*Gross appearance*	
Polypoid	33 (58.9%)
Flat or depressed	23 (41.1%)

*Histologic type*	
Well	47 (83.9%)
Moderately	8 (14.3%)
Poorly^a^	1 (1.8%)

*Lymphatic involvement (H.E and D2-40 immunohistochemistry)*	
Absent	49 (87.5%)
Present	7 (12.5%)

*Venous involvement (H.E and Victoria blue staining)*	
Absent	25 (44.6%)
Present	31 (55.4%)

*Lymph nodes metastasis*	
pN0	40 (71.4%)
ITCs (pN0i(−) and pN0i(+))	8 (14.3%)
pN1mi	3 (5.4%)
pN1	5 (8.9%)

H.E=haematoxylin and eosin staining; ITC=isolated tumour cells; s.d.=standard deviation.

a The histologic type of poorly differentiated adenocarcinoma was medullary.

**Table 2:** Tumour budding in submucosal colorectal cancers and clinicopathological features

		**Tumour budding**		
	***n***	**Present**	**Absent**	***P*-value**
Mean age (years)±s.d.	56	61.8±11.0	65.9±8.0	NS

*Gender*				
Male	41	31	10	NS
Female	15	11	4	

Size (mm)±s.d.	56	21.7±9.3	25.9±16.6	NS

*Histologic type*				
Well	47	35	12	NS
Moderately	8	7	1	
Poorly	1	0	1	

*Lymphatic involvement*				
Absent	49	35	14	0.12
Present	7	7	0	

*Lymph nodes metastasis (including ITCs and micrometastasis)*				
Absent	40	26	14	0.004
Present	16	16	0	

*Venous involvement*				
Absent	25	20	5	NS
Present	31	22	9	

ITC=isolated tumour cells; NS=not significant; s.d.=standard deviation.

**Table 3:** Predictive value of tumour budding, lymphatic invasion and combination of budding and lymphatic invasion for lymph nodes metastasis

	**Lymph node metastasis (including ITCs and micrometastasis)**		**Sensitivity (%)**	**Specificity (%)**	**Positive predictive value (%)**	**Negative predictive value (%)**
	**Present**	**Absent**				
*Lymphatic invasion*						
Present	6	1	37.5	97.5	85.7	79.6
Absent	10	39				

*Tumour budding*						
Present	16	26	100.0	65.0	38.1	100.0
Absent	0	14				

*Lymphatic invasion or tumour budding*						
Present	16	26	100.0	65.0	38.1	100.0
Absent	0	14				

*Lymphatic invasion and tumour budding*						
Present	7	0	43.7	100.0	100.0	81.6
Absent	9	40				

ITC=isolated tumour cells.
